# Tranexamic acid at cesarean delivery: drug‐error deaths

**DOI:** 10.1111/1471-0528.17292

**Published:** 2022-10-27

**Authors:** Neil F. Moran, David G. Bishop, Susan Fawcus, Edward Morris, Haleema Shakur‐Still, Adam J. Devall, Ioannis D. Gallos, Mariana Widmer, Olufemi T. Oladapo, Arri Coomarasamy, G. Justus Hofmeyr

**Affiliations:** ^1^ KwaZulu‐Natal Department of Health Durban South Africa; ^2^ Department of Obstetrics and Gynaecology University of KwaZulu‐Natal Durban South Africa; ^3^ Department of Anaesthesia University of KwaZulu‐Natal Durban South Africa; ^4^ Department of Obstetrics and Gynaecology University of Cape Town Cape Town South Africa; ^5^ Department of Obstetrics and Gynaecology Norfolk and Norwich University Hospital Norwich UK; ^6^ London School of Hygiene & Tropical Medicine London UK; ^7^ World Health Organization Collaborating Centre for Global Women's Health, Institute of Metabolism and Systems Research University of Birmingham Birmingham UK; ^8^ UNDP/UNFPA/UNICEF/WHO/World Bank Special Programme of Research, Development and Research Training in Human Reproduction, Department of Sexual and Reproductive Health and Research World Health Organization Geneva Switzerland; ^9^ Department of Obstetrics and Gynecology University of Botswana Gaborone Botswana; ^10^ Effective Care Research Unit University of the Witwatersrand Johannesburg South Africa; ^11^ Department of Obstetrics and Gynecology Walter Sisulu University Mthatha South Africa

**Keywords:** cesarean delivery, drug administration error, postpartum hemorrhage, spinal anesthesia, tranexamic acid

## Abstract

The use of tranexamic acid for postpartum hemorrhage has entered obstetrical practice globally with the evidence‐based expectation of saving lives. This improvement in the care of women with postpartum hemorrhage has come at a price. For the anesthetist, having tranexamic acid ampoules close at hand would seem an obvious strategy to facilitate its use during cesarean delivery, an important setting for severe hemorrhage. Tragically, we have identified a number of recent instances of inadvertent intrathecal administration of tranexamic acid instead of local anesthetic for spinal anesthesia. Reported cases of this catastrophic error seem to be increasing. The profound neurotoxicity of tranexamic acid causes rapid‐onset convulsions, with mortality of 50%.

How can these tragic errors be averted? Drug safety alerts have been issued by the US Food and Drug Administration and the World Health Organization, but that is not enough. We recommend extensive dissemination of information to raise awareness of this potential hazard, and local hospital protocols to ensure that tranexamic acid is stored separately from anesthetic drugs, preferably outside the operating room and with an auxiliary warning label. Implementation of safety strategies on a very large scale will be needed to ensure that the life‐saving potential of tranexamic acid is not eclipsed by drug‐error mortality.

Increasing use of tranexamic acid during cesarean delivery has been associated with fatal erroneous intrathecal administration. Tranexamic acid should not be stored on or near the theatre anaesthetic trolley.

The World Health Organization recommends tranexamic acid (1‐g) intravenously over 10 minutes, within 3 hours, for treating postpartum hemorrhage (PPH), unless contraindicated.^1^ This recommendation is based on evidence of reduced mortality compared with placebo,^2^ and has been included in national guidelines such as those in South Africa.^3^

The implementation of this recommendation, intended to improve the care of women with PPH, has exposed an unanticipated but important risk associated with increasing tranexamic acid use within obstetricalcare. The profound toxicity of intrathecal tranexamic acid was described in 1980.^4^ Tranexamic acid antagonizes inhibitory γ‐aminobutyric acid type A and glycine receptors, causing profound neuronal excitation.^5^ The typical presentation is abrupt spinal segmental myoclonus, rapidly progressing to generalized convulsions and malignant arrhythmias. Central nervous system pharmacotherapy has been advocated for treatment, and in a recent case ventriculolumbar lavage with normal saline to remove tranexamic acid from the cerebrospinal fluid was reported.^5^ A 2019 review identified 21 reported cases of inadvertent intrathecal injection of tranexamic acid since 1988, of which 20 were life‐threatening and 10 fatal.^6^ Sixteen were reported between 2009 and 2018. The increased use of tranexamic acid in the operating room to control bleeding at cesarean delivery seems to be associated with increasing numbers of inadvertent intrathecal administrations. In anticipation of its use at cesarean delivery, tranexamic acid ampoules are now frequently kept in the anesthetic drug trolley in the operating room, creating a risk of tranexamic acid being mistaken for a spinal anesthetic drug and injected intrathecally before cesarean delivery. The risk of this drug error is heightened by the similarity in shape and size of the ampoules containing tranexamic acid and those containing drugs used for spinal anesthesia (eg, bupivacaine) (Figures 1 and 2).

**Figure 1:**
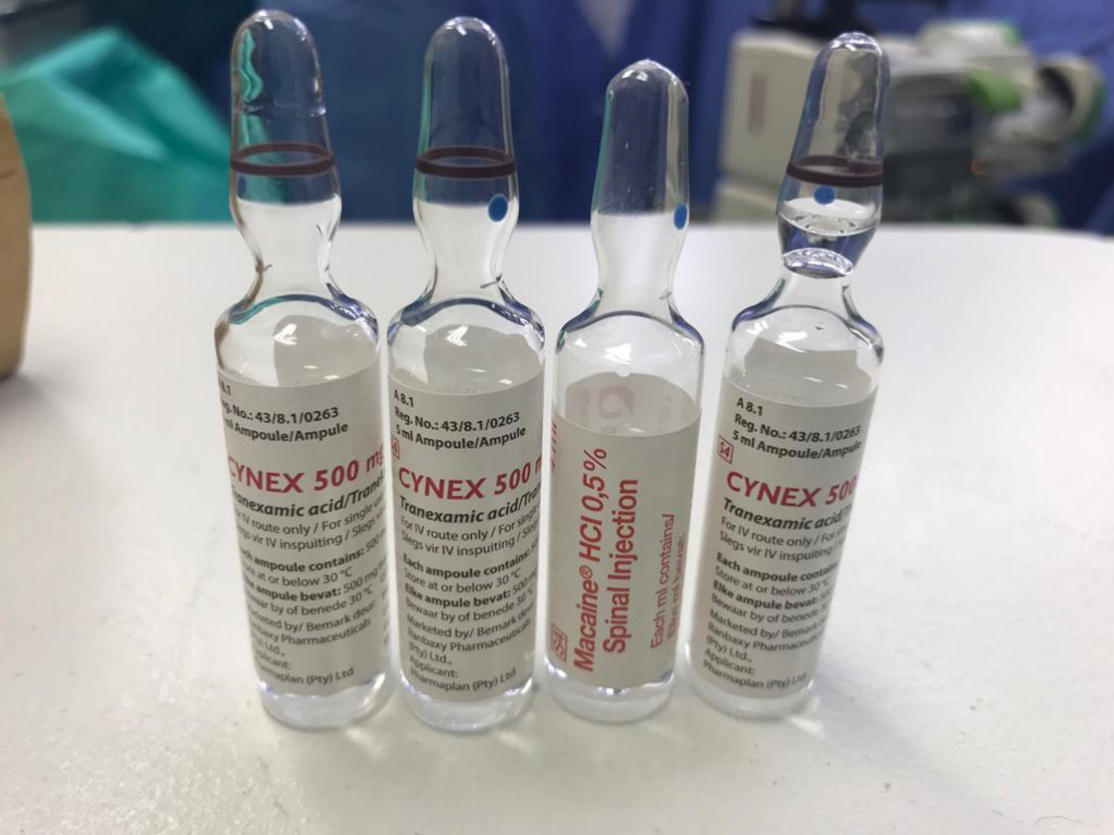
Ampoules of local anesthetic and tranexamic acid with similar appearance Moran. Tranexamic acid at cesarean delivery: drug‐error deaths. *Am J Obstet Gynecol* 2022.

**Figure 2:**
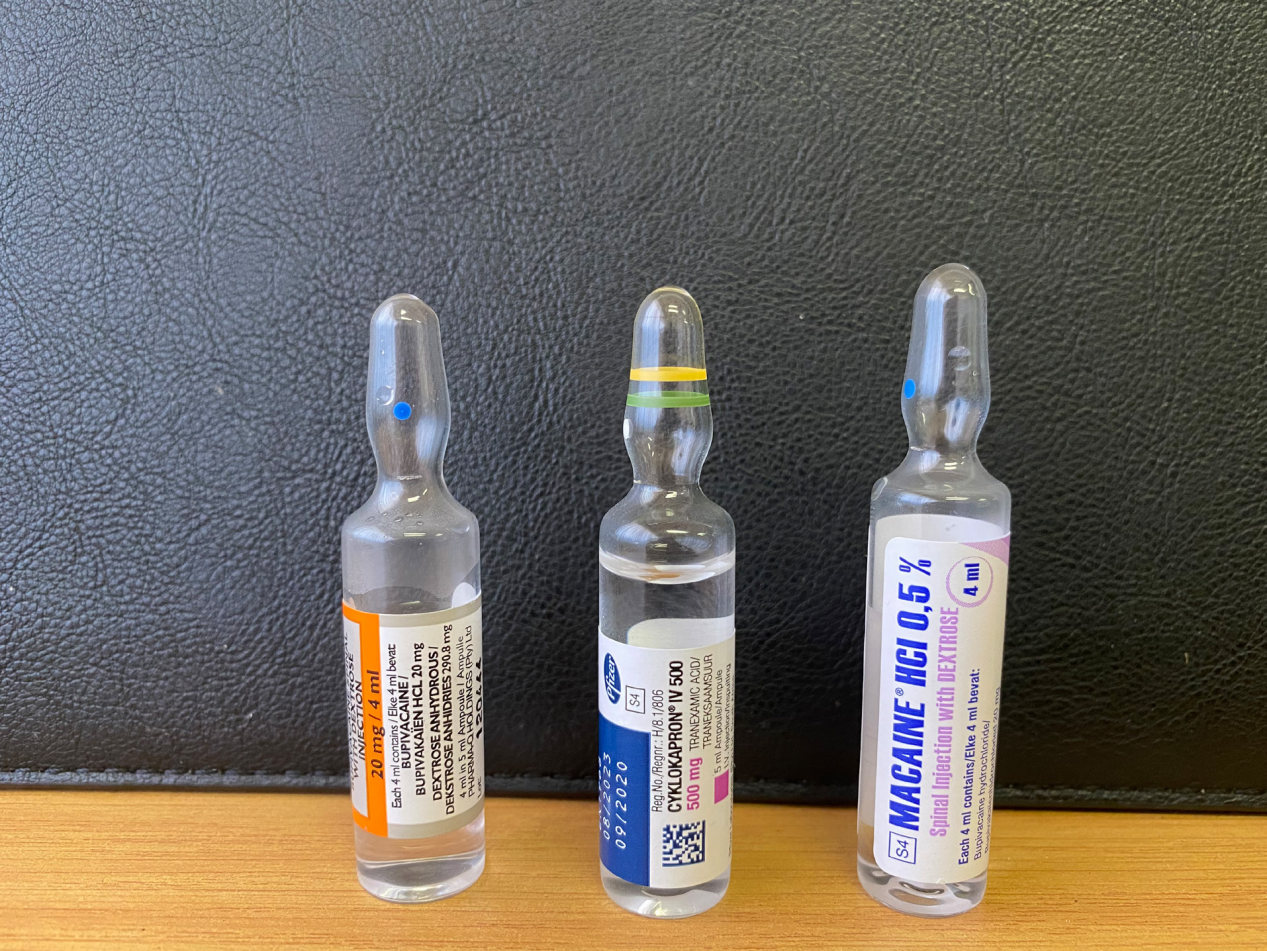
Ampoules of local anesthetic and tranexamic acid with similar size and shape Moran. Tranexamic acid at cesarean delivery: drug‐error deaths. *Am J Obstet Gynecol* 2022.

This potential drug error was highlighted, and recommendations to prevent it outlined in a 2019 publication following the identification of a fatal case through a South African confidential inquiry into maternal deaths.^7^ The authors are aware of 3 cases that occurred in South Africa during December 2021 and January 2022, raising concern that the problem may be more widespread than published case reports would suggest. Two were fatal, and one associated with prolonged ventilation and neurologic injury. Colleagues have informed us of similar instances in other countries. The true extent of the problem is difficult to measure because clinicians and managers may be reluctant to report errors that lead to major adverse outcomes. Furthermore, in some cases, the drug error may not be recognized because anesthetic practitioners may be unaware of the acute neurotoxic effects of intrathecal tranexamic acid. For example, in 1 of the 3 recent cases, the presentation of convulsions and cardiovascular instability after administration of the spinal anesthetic was ascribed to eclampsia by the attending clinicians. It was only an external review that identified the pathognomonic presentation of intrathecal tranexamic acid.

In South Africa, most specialist‐led anesthetic departments in large hospitals have well‐established protocols for drug checking during spinal anesthesia. In smaller hospitals, however, spinal anesthesia is typically administered by generalist medical officers who do not work within the structure of an anesthetic department and may not always be aware of the necessity for meticulous drug identification checks. The situation may be similar in many low‐middle–income countries, where anesthetics are often administered by a variety of healthcare cadres. Although a lack of specialist oversight may increase the risk for anesthesia‐related drug errors, drug errors owing to failure to follow guidance on the safe and secure handling of medicines can occur at any level of hospital, government‐run or private, in any part of the world.

Prophylactic use of tranexamic acid at cesarean delivery has been shown to be associated with reduced blood loss >1000 ml, but not reduced mortality or severe morbidity.^8^ Conversely, increased risk of spinal drug errors associated with routine tranexamic acid administration before cesarean delivery may outweigh any potential benefits. Policies should be informed by a broad consideration of risks and benefits, and future emerging evidence of effectiveness.

We recommend the following urgent steps to eliminate the severe and life‐threatening consequences of intrathecal administration of tranexamic acid at all hospitals where intravenous tranexamic acid is available:

1 Informing anesthetic practitioners and managers of the dangers of intrathecal tranexamic acid using various formats (scientific publications, websites for health practitioners, training programs, government‐issued circulars, local health forums for clinicians or health managers, etc.) through various channels, including:
▪ World Health Organization (Box 1)^9^
▪ National regulatory authorities (Box 1)^10^
▪ Pharmaceutical services managers
▪ Healthcare training institutions
▪ Professional societies
▪ Outreach and regional clinicians
▪ National or regional clinical guideline documents
9 Drawing up local risk management strategies and protocols for hospital health practitioners and managers related to the use of tranexamic acid, aimed at minimizing the risk of inadvertent intrathecal administration and enhancing patient safety:
▪ Tranexamic acid must always be stored separately from anesthetic drugs used in the operating room, in a secure container away from the anesthetic drugs trolley.
▪ Tranexamic acid must never be put on the spinal anesthetic trolley that is set up in preparation for administering a spinal anesthetic.
▪ Anesthetic practitioners need to carefully read the label each time before a drug for spinal anesthesia is drawn up, rather than relying on visual recognition or location of the drug ampoules.
▪ If the hospital has a look‐alike, sound‐alike (LASA) list of medications,^11^, ^12^ tranexamic acid must be added and personnel informed accordingly.
  3 Implementing the following protocols:
▪ Effective implementation of the strategies and protocols above across all hospitals in a region will require oversight with regular communication and audit of practice by a regional clinical or administrative supervisor.
▪ For countries where tranexamic acid is being introduced for use in obstetrics for the first time, it is important that the introduction incorporates information about the risk and consequences of inadvertent intrathecal administration (step 1 above) and recommendations regarding protocols to minimize risk of the drug error occurring (step 2 above).

Box 1 **World Health Organization and US Food and Drug Administration alerts regarding drug administration errors with tranexamic acid**

**World Health Organization Alert**

In 2022 the World Health Organization (WHO) issued an alert^9^: “WHO is alerting health care professionals about the risk of administration errors that can potentially occur with tranexamic acid injection. There have been reports of tranexamic acid being mistaken for obstetric spinal anesthesia used for caesarean deliveries resulting in inadvertent intrathecal administration.

Intrathecal tranexamic acid is a potent neurotoxin and neurological sequelae are manifested, with refractory seizures and 50% mortality. The profound toxicity of intrathecal tranexamic acid was described in 1980. A 2019 review identified 21 reported cases of inadvertent intrathecal injection of tranexamic acid since 1988, of which 20 were life‐threatening and 10 fatal. Sixteen were reported between 2009 and 2018.

WHO recommends early use of intravenous tranexamic acid within 3 hours of birth in addition to standard care for women with clinically diagnosed PPH following vaginal births or caesarean section. Tranexamic acid should be administered at a fixed dosage of 1 g in 10 ml (100 mg/mL) IV at 1 ml per minute, with a second dosage of 1 g IV if bleeding continues after 30 minutes.

Tranexamic acid is frequently stored in close proximity with other medicines, including injectable local anesthetics indicated for spinal analgesia (eg, for caesarean section). The presentation of some of the local anesthetics is similar to the tranexamic acid presentation (transparent ampoule containing transparent solution), which can erroneously be administer instead of the intended intrathecal anesthetic resulting in serious undesirable adverse effects.

Recently, obstetricians from several countries have reported inadvertent intrathecal tranexamic acid administration and related serious neurological injuries.

Tranexamic acid is a lifesaving medicine, however, this potential clinical risk should be considered and addressed by all operating room staff. Reviewing of existing operating room drug handling practice is required to decrease this risk, such as storage of tranexamic acid away from the anesthetic drug trolley, preferably outside the room.”

**US Food and Drug Administration Alert**

In 2020 the US Food and Drug Administration (FDA) issued an alert^10^: “The FDA is taking action to address tranexamic acid injection medication errors. This includes revising the tranexamic acid injection container labels and carton labeling to highlight the recommended intravenous route of administration; and strengthening the warnings in the tranexamic acid prescribing information to include the risk of medication errors due to incorrect route of administration.

Careful handling of tranexamic acid injection is important to prevent medication errors that could result in serious injury or death. Healthcare professionals should consider the following steps:

1. Store tranexamic acid injection vials separately from other drugs, in a way that makes the labels visible to avoid reliance on identifying drugs by the vial cap color.
2. Add an auxiliary warning label to note that the vial contains tranexamic acid.
3. Check the container label to ensure the correct product is selected and administered.
4. Utilize barcode scanning when stocking medication cabinets and preparing or administering the product.”

*PPH*, postpartum hemorrhage.

*Moran. Tranexamic acid at cesarean delivery: drug‐error deaths. Am J Obstet Gynecol 2022*.

Creative solutions are needed to ensure access for women with PPH to life‐saving therapy with tranexamic acid while guarding against the risks of mortality from drug administration errors. Regulatory bodies, suppliers, and manufacturers also have an important role in resolving this problem, for example by ensuring that tranexamic acid drug ampoules carry clear warning labels about the correct route of administration. A coordinated international effort is required to prevent inadvertent intrathecal tranexamic acid administration.

## CONFLICT OF INTERESTS

The authors report no conflict of interest.
